# Honey Fraud as a Moving Analytical Target: Omics-Informed Authentication Within a Multi-Layer Analytical Framework

**DOI:** 10.3390/foods15040712

**Published:** 2026-02-14

**Authors:** Dagmar Schoder

**Affiliations:** 1 Vétérinaires sans Frontières Austria, Veterinaerplatz 1, 1210 Vienna, Austria; dagmar.schoder@vetmeduni.ac.at or dagmar.schoder@vsf.at; 2 Centre for Food Science and Veterinary Public Health, Clinical Department for Farm Animals and Food System Transformation, University of Veterinary Medicine Vienna, Veterinaerplatz 1, 1210 Vienna, Austria

**Keywords:** honey fraud, omics-based identification, metabolomics, genomics, data-driven authentication

## Abstract

Honey fraud represents a persistent and analytically challenging form of food adulteration, driven by globalised supply chains, strong economic incentives and asymmetries in regulatory oversight and analytical capacity. Conventional physicochemical, spectroscopic and isotopic methods provide legally robust tools for routine control, yet increasingly struggle to detect sophisticated adulteration strategies that are compositionally optimised to mimic authentic honey profiles. These challenges are amplified in a global context, where heterogeneous enforcement landscapes and fragmented analytical infrastructures create exploitable vulnerabilities across international trade networks. This narrative review synthesises current knowledge on honey fraud typologies and critically evaluates established analytical approaches alongside emerging omics-based authentication strategies, including genomics, metabolomics, proteomics and microbiome profiling. Omics-based approaches extend authenticity assessment beyond single-marker paradigms by capturing multidimensional biological and compositional signatures, thereby improving sensitivity to subtle and system-aware fraud (i.e., adulteration strategies that adapt to prevailing analytical detection methods and regulatory thresholds) strategies. To maintain evidentiary clarity, this review explicitly distinguishes between analytically demonstrated vulnerabilities, technically feasible adulteration scenarios and fraud practices documented in regulatory or enforcement contexts. Advanced technology-driven strategies are therefore discussed as potential system-level risks rather than confirmed large-scale honey fraud cases. This differentiation not only safeguards evidentiary precision but also highlights the structural limits of purely analytical solutions. Beyond analytical performance, honey authentication is framed as a systemic challenge embedded in global food systems. This review highlights the need for integrated, data-driven and scalable authentication frameworks that align analytical innovation with reference harmonisation, governance structures and international regulatory cooperation to support resilient and globally robust honey authenticity control.

## 1. Introduction

Honey is one of the oldest and most culturally embedded foods consumed by humans, valued not only as a natural sweetener but also for its nutritional, medicinal and symbolic significance across civilizations [1,2]. Produced by *Apis mellifera* from diverse floral sources, honey represents a highly complex natural matrix composed of sugars, enzymes, organic acids, phenolic compounds and other bioactive constituents. Its chemical composition reflects botanical origin, geographic provenance and processing history, conferring both biological variability and analytical complexity [3,4,5]. Beyond its role as a staple food, honey occupies premium market segments associated with monofloral designation, geographical indication and health-related consumer perceptions, leading to pronounced price stratification on the global market [6].

At this scale of production and trade, honey represents a food commodity of substantial economic relevance. Total world honey production reached approximately 1.89 million tonnes in 2023, underscoring its importance within global food systems [7]. This production volume is accompanied by extensive international trade: global commerce in natural honey amounted to approximately US $2.17 billion in 2023, reflecting sustained worldwide demand and complex transnational supply chains [8]. These economic dimensions create fertile conditions for economically motivated adulteration. The pronounced price disparity between authentic honey and inexpensive sweeteners provides strong incentives for fraudulent practices at multiple points along the supply chain. Recent analyses indicate that approximately 10% of honey traded internationally may be affected by economically motivated adulteration, while targeted surveys and analytical screenings have reported adulteration signals in 30% or more of analysed samples, depending on region, sampling strategy and methodology [9].

Quantitative enforcement data on honey authenticity remain fragmented and highly context-dependent. Under the EU-coordinated border control action “From the Hives” (2021–2022), 320 consignments of imported honey were analysed using advanced analytical methods, with 147 samples (46%) flagged as suspected non-compliant with the EU Honey Directive due to the presence of added sugars [10].

Outside the EU, comparable quantitative evidence is largely confined to import surveillance. In the United States, FDA analysis of 107 imported honey samples between 2022 and 2023 identified three non-compliant products (3%) due to undeclared added sweeteners. Together, these figures illustrate that detected fraud rates are strongly shaped by regulatory scope, sampling design and analytical thresholds rather than reflecting overall market prevalence [11].

Such prevalence estimates must therefore be interpreted with caution. Many figures derive from risk-based or targeted control strategies rather than statistically representative surveys, and differences in analytical coverage and enforcement intensity further limit direct comparability across regions. While these data do not indicate uniform global prevalence, their recurrence across regulatory contexts and analytical approaches points to a structural vulnerability in honey authenticity assurance systems, supporting the view that honey fraud represents a systemic global challenge rather than isolated regulatory non-compliance.

Conventional physicochemical, spectroscopic and isotopic methods continue to form the backbone of regulatory honey authentication and remain indispensable for routine control [12,13,14]. These approaches reliably detect gross compositional deviations and classical adulteration patterns; however, they increasingly fail when fraud is subtle, compositionally optimised or deliberately engineered to mimic authentic honey profiles [15,16,17]. In this context, fraud strategies exploit not only analytical blind spots but also regulatory fragmentation and asymmetries in monitoring intensity across international supply chains.

**Figure 1:**
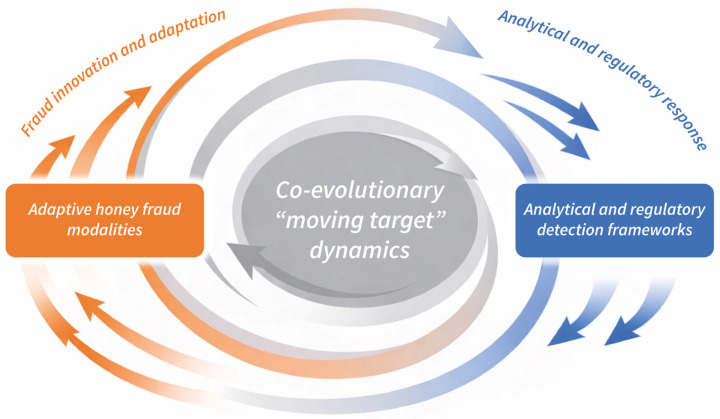
Honey fraud as a co-evolutionary “moving target”. Evolving fraud modalities and analytical and regulatory detection frameworks continuously adapt to each other, resulting in a dynamically shifting analytical landscape within globalised supply chains.

Recent evidence indicates that contemporary adulteration strategies increasingly target the analytical system itself, rather than individual quality parameters [16,17]. By fine-tuning sugar compositions, enzymatic profiles, spectral features or isotopic signatures, economically motivated adulteration can converge toward accepted authenticity ranges without triggering threshold-based detection [14,15]. These developments reflect a shift from opportunistic manipulation toward anticipatory, system-aware fraud strategies that adapt dynamically to prevailing control regimes. In a globalised market, such adaptive behaviour accelerates the co-evolution of fraud and detection, transforming honey authentication into a persistent moving target for laboratory science and regulatory governance alike [18]. This adaptive interplay between evolving honey fraud modalities and analytical and regulatory detection frameworks has transformed honey authentication into a co-evolutionary “moving target”, characterised by iterative cycles of fraud innovation and regulatory response (Figure 1).

A further, often underappreciated dimension of honey fraud relates to global disparities in analytical capacity and laboratory infrastructure. While high-income importing regions increasingly rely on advanced spectroscopic, isotopic and omics-based platforms for honey authentication, many honey-producing countries continue to depend primarily on basic physicochemical analyses, reflecting financial, technical and infrastructural constraints [19,20]. Uneven access to high-resolution instrumentation, reference materials and specialised expertise can create structural gaps within the global authenticity control network, independent of regulatory intent or compliance efforts [21]. These asymmetries highlight the need for authentication strategies that are not only analytically robust, but also scalable and adaptable across heterogeneous laboratory environments, an aspect discussed in more detail in later sections.

In response to these challenges, research over the past decade has increasingly focused on high-resolution, data-rich analytical strategies capable of capturing the biological and chemical complexity of genuine honey [5,14]. Omics-based platforms—particularly genomics, metabolomics and their integration—enable multidimensional profiling that extends beyond bulk composition and single-marker approaches. These methods offer the potential to detect inconsistencies even when adulteration is deliberately minimised or analytically camouflaged [17].

Against this background, the present review synthesises current knowledge on honey fraud typologies and critically evaluates conventional analytical approaches alongside emerging omics-based authentication strategies. Rather than focusing on individual technologies in isolation, the review emphasises system-level integration and data-driven interpretation of complex analytical evidence. The manuscript progresses from an overview of established and emerging fraud modalities (Section 2) to conventional analytical platforms (Section 3) and omics-based approaches (Section 4), before outlining governance-related perspectives and future directions for globally robust honey authentication (Section 5).

To maintain evidentiary clarity, this review distinguishes systematically between analytically demonstrated vulnerabilities, technically feasible adulteration strategies and fraud practices that are documented in regulatory or enforcement contexts. This distinction is essential to avoid conflating hypothetical risk scenarios with empirically confirmed fraud prevalence.

**Figure 2:**
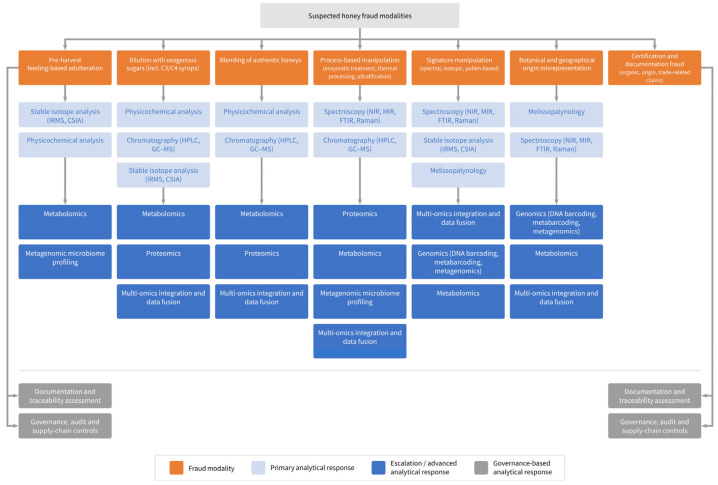
Decision tree linking honey fraud modalities to recommended analytical approaches. The schematic guides the selection of appropriate analytical strategies based on the suspected fraud modality. For certain fraud types—particularly pre-harvest feeding practices and certification or documentation fraud—governance-based controls, documentation review and traceability assessment represent the primary evidentiary layer, whereas analytical methods provide complementary or indirect support.

To guide the structured interpretation of honey fraud typologies and their corresponding analytical implications, Figure 2 presents a decision-oriented overview linking suspected fraud modalities to appropriate analytical and governance-based response pathways. The figure illustrates how different fraud scenarios necessitate distinct escalation strategies beyond routine laboratory testing.

## 2. Typology of Honey Fraud Modalities

Honey authenticity is intrinsically linked to both botanical and geographical origin, as these attributes shape compositional variability, market value and regulatory classification. Monofloral designation and geographical indication therefore represent not only quality attributes but also central vulnerability points within global honey supply chains. Fraud strategies frequently exploit origin-related expectations by mimicking botanical or geographical signatures, misusing documentation, or blending products from heterogeneous sources.

Because natural compositional variability across floral and regional origins often overlaps with analytical reference ranges, origin-related misrepresentation is inherently difficult to resolve by laboratory analysis alone. These characteristics make botanical and geographical origin a recurring axis of both established and emerging honey fraud modalities, providing an essential contextual framework for the typology outlined below [5].

This section structures honey fraud modalities into established and emerging categories to reflect differences in enforcement relevance, analytical feasibility and evidence level, as summarised in Table 1 and Table 2. Honey fraud encompasses a heterogeneous set of practices that differ in their mechanistic basis, strategic intent and susceptibility to analytical detection. While early forms of adulteration primarily targeted bulk composition and easily accessible quality markers, contemporary fraud increasingly exploits analytical blind spots and system-level vulnerabilities. To reflect this evolution, the fraud modalities described in the scientific literature and regulatory casework are structured here into two complementary tiers: established modalities that underpin current enforcement frameworks (Table 1 and Section 2.1) and emerging, technology-driven extensions that challenge these frameworks (Table 2 and Section 2.2). Figure 2 provides a complementary decision-oriented perspective by mapping these fraud modalities to their respective analytical and governance-based response pathways.

**Table 1:** Established honey fraud modalities addressed by conventional analytical and regulatory frameworks.

Fraud Modality	Operational Description	Strategic Motivation	Limitations	Market Impact	Literature Basis
Pre-harvest feeding-based adulteration	Feeding of sugar syrups to *Apis mellifera* colonies or feeding of *Apis mellifera* honey to *Apis cerana cerana* to generate honey independent of natural nectar foraging	Circumvention of nectar availability, production cost reduction, exploitation of premium origin narratives	Largely invisible to conventional compositional analysis; biochemical compliance despite violated production authenticity	Market distortion through misrepresentation of origin, species-specific value inflation	[4,5,6,22,23]
Dilution with exogenous sugars (incl. C3/C4 syrups)	Addition of external sugar syrups to increase volume while maintaining acceptable sensory properties	Evasion of compositional thresholds	Increasing convergence with authentic sugar profiles reduces sensitivity of physicochemical, spectroscopic and chromatographic screening	High-volume adulteration with systematic price depression and erosion of consumer trust	[14,24,25]
Blending of authentic honeys	Mixing honeys of different origins or qualities to mask atypical or low-quality lots	Stabilisation of analytical and sensory profiles	Profile averaging undermines isotope ratios, chromatographic markers and multivariate discrimination	Quality downgrading marketed as premium product, distortion of origin-linked pricing	[5,26]
Process-based manipulation (enzymatic treatment, thermal processing, ultrafiltration)	Technical processing to alter enzymatic activity, sugar composition or pollen content	Restoration of regulatory compliance indicators	HMF, diastase and pollen-based criteria can be manipulated without altering the core honey matrix	Enables regulatory-compliant appearance of low-quality or adulterated honey; shifts enforcement burden	[23,27,28]
Signature manipulation (spectral, isotopic, pollen-based)	Targeted modification of analytical markers to mimic authentic reference ranges	Circumvention of marker-based authentication	Exploits reliance on fixed thresholds and reference databases across IRMS, spectroscopy and melissopalynology	Increased false negatives, escalation of analytical costs and intensification of the fraud–detection arms race	[4,29,30]
Botanical and geographical origin misrepresentation	False declaration of floral or geographical origin without substantial matrix alteration	Market value inflation	Analytical confirmation is limited by natural variability and overlapping reference ranges	Premium capture without corresponding production costs; damage to protected origin markets and honest producers	[4,31]
Certification and documentation fraud (organic, origin, trade-related claims)	Misuse or falsification of certification, traceability or customs documentation	Regulatory and tariff circumvention	Largely inaccessible to analytical testing; requires governance, audit and supply chain controls	Systemic market distortion and erosion of trust in certification and traceability schemes	[4,29]

**Table 2:** Emerging and technology-driven extensions of established honey fraud modalities.

Emerging Fraud Modality *	Operational Description	Strategic Motivation	Limitations	Market Impact	Current Implementation Status	Evidence Basis
AI-optimised designer blends	Algorithm-guided blending of syrups and authentic honeys to optimise conformity across multiple analytical parameters	Optimisation against multi-parameter detection	Undermines single-marker, threshold-based and multivariate models through engineered convergence	Scalable, hard-to-detect adulteration with potential for large-scale market penetration and price distortion	Technically feasible	[5,32,33,34]
Targeted enzymatic conversion systems	Controlled enzymatic modification of sugars to approximate authentic compositional patterns	Anticipatory alignment with analytical criteria	Challenges chromatographic and enzymatic authenticity parameters; masking of adulteration markers	Enables systematic circumvention of established analytical criteria; moderate but growing relevance	Analytically vulnerable	[35,36,37]
Advanced spectral and isotopic mimicry	Coordinated manipulation of multiple analytical signatures using feedback from detection systems	Evasion of adaptive analytical frameworks	Reduces robustness of IRMS, spectroscopy and hybrid authentication workflows	Escalates analytical arms race and enforcement costs without immediate mass-market dominance	Analytically vulnerable	[6,38,39]
Digital and traceability system manipulation	Exploitation of digital certification, blockchain or documentation infrastructures	Systemic governance evasion	Shifts fraud beyond laboratory detection into cyber-physical and administrative domains	System-level distortion of trade, certification and consumer trust	Documented enforcement cases	[10,40,41]

* Table 2 provides a representative, non-exhaustive overview of emerging and technology-driven extensions of established honey fraud modalities, selected to illustrate current and anticipated strategic developments rather than to enumerate all possible variants.

### 2.1. Established Honey Fraud Modalities

Established honey fraud modalities comprise a set of practices that are consistently documented in regulatory casework and extensively analysed in the scientific literature. Comprehensive reviews by Sotiropoulou et al. [12], Faustino and Pinheiro [13] and Nyarko et al. [42] converge in showing that these modalities involve direct intervention in honey composition, processing parameters or documentation. Together, they form the empirical foundation upon which current authentication standards and routine control strategies have been developed. Table 1 provides a structured overview of established honey fraud modalities discussed in this section.

#### 2.1.1. Pre-Harvest Feeding Practices as Adulteration Modalities

Beyond post-harvest compositional manipulation, certain adulteration practices intervene upstream at the level of bee feeding and honey production itself. One such practice involves the intentional feeding of sugar syrups to *Apis mellifera* colonies during nectar flow, resulting in honey that is biochemically derived from exogenous sugars rather than floral nectar. While such feeding may be permitted under specific husbandry conditions, its deliberate use to generate marketable honey constitutes a form of adulteration, as the resulting product no longer reflects the botanical and biochemical characteristics implied by honey authenticity standards [4,5,6].

A related practice concerns the feeding of *Apis mellifera* honey to *Apis cerana cerana* colonies to produce so-called “Apis cerana honey”. Although marketed as a distinct and premium product, this approach replaces natural nectar foraging with secondary honey conversion, thereby decoupling the final product from its declared botanical and ecological origin [22,23]. From an authenticity perspective, both practices represent biologically mediated forms of adulteration that challenge conventional analytical paradigms, as the resulting honey may remain compositionally compliant while violating origin and production integrity expectations (see also Table 1).

#### 2.1.2. Matrix-Altering Adulteration and Compositional Manipulation

The most prevalent and intensively studied fraud modality is dilution with exogenous sugars, including C3 and C4 plant-derived syrups. Reviews and original studies consistently report that this practice aims to increase volume while maintaining acceptable sensory properties and apparent regulatory compliance [14,42]. Method-focused studies demonstrate that increasing convergence between adulterated and authentic sugar profiles progressively reduces the sensitivity of physicochemical screening, chromatographic fingerprinting and spectroscopic approaches, particularly under threshold-based decision frameworks [24,25]. As highlighted by Sotiropoulou et al. [12], this convergence effect represents a core analytical vulnerability in routine honey control and explains why sugar dilution remains both analytically challenging and economically impactful. This regulatory relevance is reflected in official EU control data focusing on sugar-based adulteration. An earlier EU-coordinated honey authenticity control plan (2015–2017) analysed 893 honey samples submitted for confirmatory testing after national screening, of which 14% were confirmed to contain added sugars. Although not based on random sampling, these data illustrate the repeated detection of matrix-altering compositional adulteration across multiple stages of the supply chain rather than indicating overall market [43].

A closely related modality is the blending of authentic honeys of different origins or qualities. Blending exploits the natural compositional variability of authentic honeys by mixing products with different analytical profiles. As demonstrated by Seraglio et al. [26], such mixing can dilute atypical or low-quality characteristics, resulting in averaged profiles that fall within expected reference ranges without the addition of foreign substances. This reduces the discriminatory power of isotope ratio analysis, chromatographic fingerprints and multivariate classification models. These findings are synthesised in broader authenticity reviews [12,29], which emphasise that blending poses a particular analytical challenge because it operates entirely within the compositional space of genuine honeys while enabling downgraded products to be marketed as premium or origin-specific.

Another well-established category comprises process-based manipulation, including enzymatic treatment, thermal processing and ultrafiltration. Multiple studies document how such interventions are used to modify enzymatic activity, sugar composition or pollen content in order to restore compliance with regulatory indicators such as hydroxymethylfurfural concentration, diastase activity or pollen thresholds [23,27,28]. Reviews of honey quality control note that these processing steps can substantially alter key authenticity markers without fundamentally changing the overall honey matrix [12,13], thereby complicating enforcement strategies based on single-parameter criteria.

#### 2.1.3. Signature- and Marker-Based Manipulation Strategies

Beyond compositional and processing interventions, established fraud modalities also include signature manipulation, targeting analytical markers themselves. Studies reviewed by Bobis et al. [4] and Li et al. [30] describe how spectral, isotopic and pollen-based signatures may converge with authentic reference ranges under certain conditions. As discussed in the broader fraud literature [29], reliance on fixed thresholds and static reference databases across IRMS, vibrational spectroscopy and melissopalynology increases the risk of false-negative outcomes, particularly when multiple markers are interpreted independently rather than in an integrated framework.

#### 2.1.4. Botanical and Geographical Origin Misrepresentation

Misrepresentation of botanical and geographical origin constitutes another extensively documented fraud practice. Reviews by Geană et al. [31] and Bobis et al. [4] show that false origin claims are frequently made without inducing substantial changes in the honey matrix. Analytical confirmation is inherently constrained by natural compositional variability and overlapping reference ranges, especially for botanically or climatically similar regions. As a result, this modality primarily enables market value inflation and undermines protected designation schemes, rather than exploiting analytical blind spots in bulk composition.

#### 2.1.5. Certification- and Documentation-Based Fraud

Finally, certification and documentation fraud encompasses the misuse or falsification of organic labels, origin certificates and trade-related documentation. As emphasised in reviews by Kaldeli et al. [29] and Bobis et al. [4], these practices are largely inaccessible to laboratory-based analytical testing and instead require governance-, audit- and supply chain-oriented controls. Their continued occurrence highlights the structural limitations of analytical authentication when applied in isolation and underscores the complementary role of non-analytical enforcement mechanisms.

### 2.2. Emerging and Technology-Driven Fraud Modalities

Beyond established forms of honey adulteration that directly alter honey composition, recent scientific and regulatory literature has drawn attention to technology-enabled extensions of fraud logics that primarily challenge analytical frameworks, governance structures and system-level controls rather than introducing entirely new adulteration practices. The fraud modalities summarised in Table 2 therefore do not represent a homogeneous class of newly emerging honey fraud schemes. Instead, they reflect heterogeneous extensions of known mechanisms whose analytical feasibility, empirical documentation and regulatory relevance differ substantially. Table 2 summarises emerging and technology-driven fraud extensions and supports the subsection-based discussion in Section 2.2.

#### 2.2.1. AI-Optimised Designer Blends

AI-optimised designer blends (i.e., deliberately formulated mixtures optimised to meet analytical acceptance criteria) describe algorithm-guided formulation strategies in which authentic honey and exogenous sugar components are combined in a way that minimises detectable deviations across multiple analytical dimensions simultaneously. This category explicitly does not imply documented honey fraud cases but captures the analytical feasibility of multivariate optimisation against existing detection frameworks.

A growing body of analytical and methodological literature demonstrates that machine-learning and chemometric optimisation approaches can engineer convergence across complex physicochemical parameter spaces in food matrices. Reviews on honey authenticity and multivariate detection limits highlight that modern authentication increasingly relies on correlated marker sets rather than single parameters, thereby introducing structural vulnerabilities when optimisation strategies are applied [5,6]. Chemometric reviews further demonstrate how algorithm-guided feature selection and optimisation can exploit these correlations, particularly in data-rich compositional systems [32].

Honey-specific applications of machine learning underscore both the power and fragility of such approaches. FT-based machine-learning models have been shown to achieve high classification accuracy for botanical and geographical origin discrimination [33], while supervised learning models successfully detect syrup adulteration under controlled conditions [34]. Collectively, these studies demonstrate analytical feasibility in principle, while simultaneously revealing that performance is strongly dependent on training data structure and parameter selection [29].

At present, AI-optimised designer blends must therefore be classified as technically feasible but not enforcement-documented in honey. Their relevance in Table 2 lies in exposing structural vulnerabilities of multivariate authentication systems, rather than in evidencing established fraudulent practice.

#### 2.2.2. Targeted Enzymatic Conversion Systems

Targeted enzymatic conversion systems encompass the controlled use of enzymatic pathways to modify carbohydrate profiles in a manner that approximates authenticity-relevant compositional patterns. This category explicitly excludes natural enzymatic activity intrinsic to honey and bee metabolism and instead focuses on the potential misuse of legitimate enzymatic technologies.

Enzymatic modification of sugars is well characterised in honey biochemistry and food biotechnology. A recent study demonstrates that enzyme-driven transformations can alter honey sugar composition and related analytical markers under defined conditions [35]. Beyond honey-specific systems, controlled enzymatic pathways capable of reshaping carbohydrate profiles are extensively documented in molecular biosciences, illustrating the technical plausibility of targeted sugar modification [36].

Reviews on honey quality and biological functionality further contextualise enzymes as critical contributors to both compositional and functional properties of honey, without framing these processes as fraudulent per se [23,37]. Importantly, this literature demonstrates that enzymatic technologies routinely applied in legitimate food processing can influence markers that are also used for authenticity assessment. Despite clear biochemical feasibility, targeted enzymatic conversion has not yet been explicitly documented in the honey fraud literature as a confirmed, stand-alone adulteration practice. Accordingly, this category is best classified as indirectly implementable, with relevance primarily for analytical interpretation and regulatory vigilance, rather than as an established fraud strategy.

#### 2.2.3. Advanced Spectral and Isotopic Mimicry

Advanced spectral and isotopic mimicry refers to coordinated manipulation of compositional attributes that collectively reduce the discriminatory power of spectroscopic and isotopic authentication methods. This category does not imply the existence of dedicated “mimicry technologies” but captures empirically documented analytical overlap and sensitivity limitations.

Strong evidence for such vulnerabilities is provided by official EU control data. The Joint Research Centre of the European Commission reports that coordinated compositional convergence (i.e., the deliberate engineering of adulterated honey to mimic the compositional profile of authentic honey) can lead to overlap between authentic and adulterated honeys within accepted analytical tolerances, particularly for LC-IRMS and vibrational spectroscopy [38]. These findings are reinforced by methodological studies demonstrating intrinsic limits of spectroscopic discrimination in complex food matrices [6].

Food-specific studies further document that the robustness of isotopic and spectroscopic markers decreases as adulteration strategies become more sophisticated, particularly when multiple parameters are adjusted concurrently [39,44]. Importantly, these studies provide empirical evidence of analytical vulnerability, not proof of routine or large-scale fraudulent implementation. Consequently, advanced spectral and isotopic mimicry is best characterised as analytically demonstrated and enforcement-relevant, while remaining only indirectly implemented in honey fraud contexts.

#### 2.2.4. Digital and Traceability System Manipulation

Digital and traceability system manipulation represents the fraud modality with the clearest empirical grounding among the categories listed in Table 2. It encompasses the exploitation of digital certification, documentation and traceability infrastructures rather than manipulation of honey composition itself.

Governance and information-systems literature identifies structural weaknesses in digital governance models, including trust assumptions, data-integrity dependencies and scalability challenges [40]. Blockchain-based traceability systems, while often presented as fraud-resistant, are similarly constrained by “garbage-in–garbage-out” dynamics and governance blind spots [41,45].

Crucially, official reports from the European Commission DG SANTE document recurrent documentation- and traceability-related irregularities in the honey trade during coordinated control actions, confirming that this modality is documented in enforcement practice [10]. Unlike analytically focused fraud strategies, digital manipulation operates largely beyond the reach of laboratory-based controls and directly undermines regulatory oversight and consumer trust.

#### 2.2.5. Synthesis

Taken together, the fraud modalities summarised in Table 2 reflect heterogeneous evidence levels rather than a uniform emergence of new honey fraud practices. Digital and traceability system manipulation is supported by direct enforcement evidence with clear market relevance. Advanced spectral and isotopic mimicry is empirically supported as an analytical vulnerability, while AI-optimised designer blends and targeted enzymatic conversion systems remain technically feasible but empirically unconfirmed for honey.

Accordingly, Table 2 should be interpreted as a risk- and vulnerability-oriented framework rather than a catalogue of established fraud schemes. Its primary function is to delineate where current honey authentication systems are analytically challenged, structurally exposed or governance-limited, thereby providing the conceptual basis for the analytical strategies discussed in subsequent sections. These heterogeneous extensions should therefore be interpreted within the broader co-evolutionary context between fraud innovation and analytical and regulatory response, illustrated in Figure 1.

## 3. Conventional Analytical Approaches for Honey Authentication: Capabilities and Limitations

**Figure 3:**
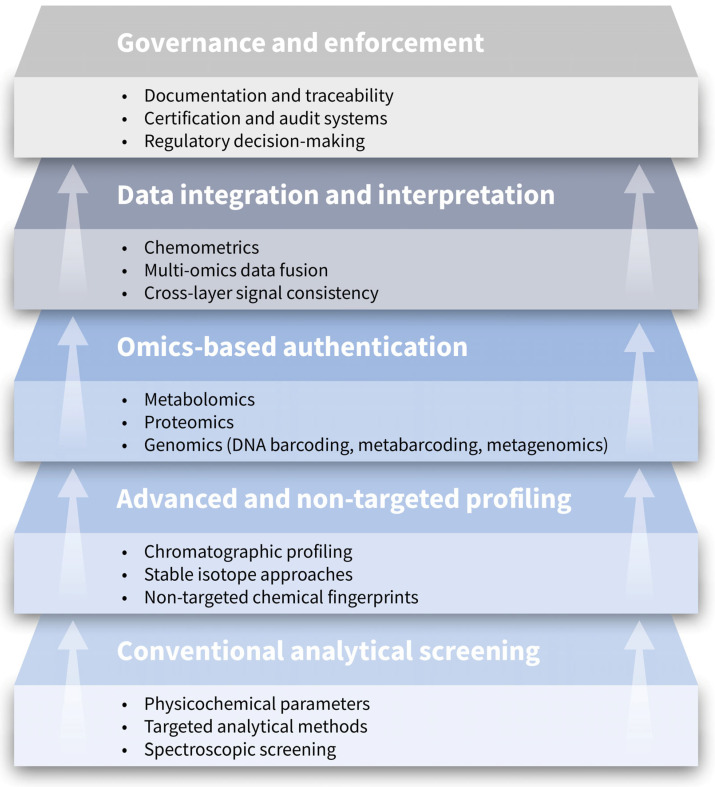
Multi-layer authentication framework for honey fraud detection and control. The framework integrates conventional screening, advanced profiling, omics-based authentication and cross-layer data integration to support robust interpretation and governance-level decision-making.

This section reviews conventional analytical approaches for honey authentication, with Table 3 providing a structured overview of method principles, capabilities and limitations. The analytical approaches discussed in this section can be conceptually situated within a multi-layer authentication framework that integrates conventional screening, advanced profiling, omics-based methods and cross-layer data interpretation (Figure 3). This framework highlights how analytical evidence is progressively consolidated to support robust interpretation and enforcement-relevant decision-making.

Conventional analytical approaches remain the backbone of honey authentication because they provide quantifiable, reproducible and legally defensible evidence within established regulatory frameworks. Their central role reflects an adulteration landscape that, for decades, was dominated by bulk compositional manipulation and readily detectable quality deviations. Comprehensive reviews consistently show that physicochemical analysis, spectroscopy, chromatography, stable isotope analysis and melissopalynology form the core of routine control strategies worldwide [5,12,13].

However, contemporary honey fraud increasingly exploits natural compositional variability, targeted processing and analytical convergence, rather than producing overt deviations from regulatory thresholds. As a result, the discriminatory performance of conventional methods now depends strongly on calibration design, dataset representativeness and interlaboratory harmonisation—parameters that vary substantially across studies and control systems [6,15]. This section critically examines the capabilities and structural limitations of established analytical platforms, as summarised in Table 3, in relation to contemporary fraud strategies.

While several conventional analytical methods increasingly rely on chemometric and multivariate data analysis, these approaches remain conceptually distinct from omics-based profiling, as they operate on established physicochemical, spectroscopic or isotopic measurements rather than on high-dimensional biological data layers. Table 3 summarises conventional analytical approaches for honey authentication discussed in this section.

**Table 3:** Overview of established analytical methods for honey authentication.

Analytical Method *	Analytical Focus	Strengths	Limitations	Relevance	Literature Basis
Physicochemical Analysis	Moisture, pH, EC, HMF, enzyme activity, diastase activity, free acidity	Simple, low-cost, regulatory familiarity; first-line screening	High natural variability; threshold-based criteria easily bypassed; subtle adulteration undetected	Increasingly insufficient as fraud targets compositional convergence and enzymatic manipulation	[4,12,13,26,46,47,48]
Spectroscopy (NIR, MIR, FTIR, Raman)	Global spectral fingerprints; classification of floral and geographical origin; detection of adulteration-related spectral deviations	Rapid, non-destructive, scalable; minimal sample preparation; good for screening	Strong dependence on preprocessing; poor inter-lab reproducibility; model fragility; instrumental heterogeneity	Sensitive to engineered spectral similarity, dataset drift, low-level adulteration	[5,12,15,24,45,49,50,51]
Chromatography (HPLC, GC–MS)	Sugars, organic acids, amino acids, phenolics, volatiles	High molecular specificity; quantitative precision; confirmatory method	Labour-intensive; expensive; limited throughput; partial overlap between authentic and adulterated profiles	Fraud increasingly exploits convergence within authentic ranges; volatile fingerprint manipulation	[5,16,49,50,52,53]
Stable Isotope Analysis (IRMS, CSIA)	δ^13^C (bulk and compound-specific), isotopic composition of sugars, amino acids	High evidentiary robustness; strong regulatory acceptance; discriminates C3/C4 signatures	Climate-driven variability; engineered isotopic mimicry; limited scalability; costly instrumentation	Effective for classical syrup adulteration; vulnerable to sophisticated blending strategies	[14,25,30]
Melissopalynology	Pollen identification; botanical/geographical origin	Low-cost; well-established; infrastructure-light; interpretability	Pollen removal or supplementation; natural variability; low sensitivity to biochemical fraud; limited as a standalone method	Increasingly unreliable when pollen is manipulated; requires integration with complementary analytical approaches	[23,27,28,51]

* Table 3 provides a representative overview of established analytical approaches for honey authentication, selected based on regulatory relevance and methodological maturity rather than as an exhaustive literature survey.

### 3.1. Physicochemical Analysis

Physicochemical parameters—including moisture content, pH, electrical conductivity, hydroxymethylfurfural (HMF), diastase activity and free acidity—constitute the regulatory foundation of honey authentication due to their simplicity, low cost and long-standing standardisation. Reviews by Faustino & Pinheiro [13] and Sotiropoulou et al. [12] highlight that these parameters were historically effective because early adulteration produced sufficiently large compositional shifts to trigger threshold-based criteria.

Recent primary studies, however, consistently demonstrate intrinsic limitations. Natural variability driven by botanical origin, climate, storage and processing frequently overlaps with values observed in adulterated samples [4,46]. Low-level syrup additions may leave moisture, pH and free acidity unchanged, while controlled thermal or enzymatic processing can partially restore diastase activity or limit HMF accumulation, thereby masking prior degradation or adulteration [27,28,49].

As a consequence, physicochemical analysis has increasingly shifted from a confirmatory role to a first-line screening function. While still indispensable for regulatory compliance assessment, its ability to resolve subtle or compositionally convergent fraud scenarios is limited unless combined with orthogonal analytical techniques [12,13]. This limitation is particularly relevant in global control settings where physicochemical testing remains the primary or sole analytical tool.

### 3.2. Spectroscopic Methods

Spectroscopic techniques—including near-infrared (NIR), mid-infrared (MIR/FTIR) and Raman spectroscopy—are widely applied for rapid, non-destructive screening of honey authenticity. Reviews by Zhang et al. [5], Sotiropoulou et al. [12] and Mendes and Duarte [15] emphasise their operational advantages: minimal sample preparation, high throughput and suitability for routine monitoring.

Multiple studies confirm that spectroscopic approaches, particularly when combined with chemometrics, can detect moderate syrup adulteration and differentiate honey types under controlled conditions [24,49]. UV–Vis spectroscopy and fluorescence-based methods further extend this screening toolbox, especially in resource-limited settings [31,54].

At the same time, the literature consistently highlights structural vulnerabilities. Spectroscopic performance is highly sensitive to preprocessing choices, instrument-specific variability and calibration dataset composition. Studies assessing interlaboratory transferability show marked declines in classification accuracy when models are applied outside their original calibration domain [52,55]. Moreover, emerging fraud strategies increasingly aim at spectral convergence, generating fingerprints that fall within authentic variability ranges and thereby erode sensitivity at low adulteration levels [5,24].

Consequently, spectroscopic methods are best interpreted as highly efficient screening tools rather than standalone confirmatory techniques. Their reliability in contemporary fraud contexts depends critically on continuous model updating, representative reference datasets and integration with complementary analytical platforms.

### 3.3. Chromatographic Methods

Chromatographic techniques, particularly high-performance liquid chromatography (HPLC) and gas chromatography coupled to mass spectrometry (GC–MS), have long served as confirmatory tools in honey authentication due to their molecular specificity and quantitative precision. Comprehensive reviews consistently highlight their ability to resolve sugars, organic acids, amino acids, phenolic compounds and volatile profiles, thereby providing detailed compositional insight beyond bulk screening methods [5,16].

Multiple studies demonstrate that chromatographic profiling can reliably detect syrup adulteration and atypical compositional patterns under controlled conditions. HPLC-based sugar profiling and chemometric fingerprinting have been shown to differentiate authentic honeys from samples adulterated with common sugar syrups [16,26]. Similarly, GC–MS analyses of volatile and semi-volatile compounds contribute to floral and geographical characterisation and can reveal deviations associated with non-authentic processing or blending [53]. Reviews synthesising these findings emphasise that chromatographic approaches remain among the most information-rich analytical platforms available for honey authenticity assessment [52].

At the same time, the literature clearly documents structural limitations. Contemporary adulteration strategies increasingly exploit compositional convergence, whereby engineered syrups or blended products generate chromatographic profiles that fall within the natural variability of authentic honeys. Reviews note that this overlap can substantially weaken discrimination when reference datasets are insufficiently representative or geographically constrained [5]. A recent study further shows that even high-resolution chromatographic fingerprints may fail to resolve low-level adulteration when marker compounds are selectively matched or diluted [26].

Operational constraints also limit the scalability of chromatographic methods in routine control. Reviews and empirical assessments highlight high analytical costs, labour-intensive workflows, specialised instrumentation requirements and limited throughput as persistent barriers, particularly in high-volume screening contexts [16,50]. In addition, interlaboratory harmonisation remains challenging due to differences in sample preparation, chromatographic conditions and data processing, which can compromise comparability across control systems [52].

Taken together, chromatographic methods retain a central confirmatory role in honey authentication, particularly for targeted investigation and legal substantiation. However, converging evidence indicates that their standalone discriminatory power is increasingly constrained by compositional overlap and adaptive fraud strategies. As reflected in Table 3, their contemporary value lies primarily in integration with orthogonal analytical platforms, where molecular specificity can complement broader screening and system-level assessment rather than serve as an isolated solution.

### 3.4. Stable Isotope Analysis (IRMS, CSIA)

Stable isotope analysis, particularly isotope ratio mass spectrometry (IRMS) and compound-specific isotope analysis (CSIA), is widely regarded as one of the most legally robust and conceptually grounded approaches for honey authentication. Reviews consistently highlight that IRMS provides direct evidence on carbon assimilation pathways and is therefore especially effective for detecting adulteration with C4 plant-derived sugars, which differ isotopically from the C3 plant sources that dominate natural nectar [14,44].

Recent studies demonstrate that bulk δ^13^C measurements reliably identify classical C4-syrup adulteration and feed-derived dilution under controlled conditions [24,25]. CSIA further extends this capability by resolving isotopic signatures at the level of individual sugars or amino acids, thereby improving discrimination when bulk isotope ratios overlap with authentic ranges [30]. Reviews synthesising these findings emphasise that CSIA represents a significant methodological advance, particularly in complex adulteration scenarios involving mixed sugar sources [44].

Despite these strengths, the literature also documents emerging vulnerabilities. Several studies note that climate-driven variability, regional differences in plant isotopic baselines and shifts in agricultural practices can influence δ^13^C reference ranges, complicating interpretation in geographically diverse datasets [44,47]. In addition, blending strategies that combine honeys from different origins or carefully selected syrup sources can partially reproduce authentic isotopic signatures, thereby reducing discriminatory power at low adulteration levels [29].

Operational and structural constraints further limit the universal applicability of isotope-based methods. Reviews consistently highlight high instrumentation costs, specialised expertise requirements and limited throughput as barriers to routine deployment, particularly outside well-resourced regulatory laboratories [14,45]. Moreover, interpretation depends critically on the availability and representativeness of reference databases, as well as on harmonised analytical protocols across laboratories [44].

Taken together, stable isotope analysis remains a cornerstone of honey authentication, particularly for classical syrup adulteration and legally defensible confirmation. However, converging evidence indicates that its effectiveness is increasingly context-dependent, with vulnerabilities arising from isotopic baseline variability and adaptive blending strategies. As reflected in Table 3, IRMS and CSIA are most effective when embedded within integrated analytical frameworks, where isotopic evidence is interpreted alongside complementary compositional and spectroscopic data rather than as a standalone determinant of authenticity.

### 3.5. Melissopalynology

Melissopalynology, defined as the microscopic identification and quantification of pollen grains in honey, represents one of the earliest systematic approaches to honey authentication and remains primarily associated with the verification of botanical origin. The method is conceptually grounded in the assumption that pollen spectra reflect floral sources visited by bees during nectar collection. In contrast to compositional or isotopic techniques, melissopalynology does not interrogate the chemical matrix of honey directly but relies on biological particulates embedded within the product [23,51].

Available analyses show that pollen examination can support botanical attribution under well-defined and restricted conditions, particularly for monofloral honeys characterised by distinctive and abundant pollen markers. In such cases, pollen spectra may corroborate declared floral origin when interpreted by experienced analysts using region-specific reference collections [28,51]. Under these circumstances, melissopalynology contributes an independent line of evidence that complements physicochemical and chromatographic data rather than replacing them.

However, several constraints limit the general reliability of pollen-based authentication. Pollen representation in honey is influenced by multiple factors beyond floral origin, including differences in pollen production among plant species, bee foraging behaviour and variability in pollen transfer from nectar to honey. These factors can lead to substantial variation in pollen abundance and composition even among authentic honeys derived from the same botanical source [23,51]. As a result, quantitative interpretation of pollen counts is inherently uncertain and difficult to standardise across products and regions.

Methodological limitations further constrain the approach. Melissopalynology is intrinsically semi-quantitative and operator-dependent, relying on microscopic identification and expert judgement. Analytical outcomes are sensitive to analyst experience, reference library completeness and classification criteria, which complicates interlaboratory comparability and reproducibility [28]. This interpretative dependence distinguishes pollen analysis from instrumental techniques, where measurement uncertainty can be more readily quantified and harmonised.

Processing effects represent an additional limitation with direct relevance for fraud assessment. Filtration and ultrafiltration can substantially reduce pollen content without materially altering the chemical composition of honey. Under such conditions, reduced or absent pollen cannot be interpreted as evidence of fraudulent origin, but it nevertheless renders melissopalynological evaluation inconclusive [27,28]. This decoupling of pollen signatures from origin claims weakens the method in contemporary supply chains where filtered honeys are widely traded.

From an authentication perspective, melissopalynology is particularly vulnerable to processing and compositional averaging practices rather than to deliberate analytical evasion. Blending honeys of different origins or selectively filtering products can yield pollen profiles that are ambiguous or misleading while remaining compliant with other analytical criteria [27,51]. These practices exploit the limited scope of pollen analysis when applied as a decisive criterion for authenticity.

In this context, reclassification based predominantly on pollen evidence conflicted with physicochemical and phytochemical characteristics, highlighting the risks of over-interpreting melissopalynological findings when detached from complementary analytical data [27]. Related analyses similarly indicate that pollen data alone are insufficient to resolve complex authenticity questions dominated by processing, blending or biochemical manipulation [23,28].

Taken together, the available evidence delineates a clearly bounded role for melissopalynology in contemporary honey authentication. The method retains value for targeted botanical origin assessment, particularly in expert-driven evaluations of monofloral honeys. At the same time, its reliability is strongly context-dependent and constrained by biological variability, processing effects and interpretative subjectivity. Consequently, pollen analysis is most appropriately applied as a supportive component within integrated authentication frameworks, where its findings are interpreted alongside compositional, chromatographic and isotopic evidence rather than used as a standalone determinant of honey authenticity. In practical expert assessment, melissopalynological interpretation is rarely based on pollen analysis alone. Experienced analysts routinely integrate additional contextual information, including declared geographical origin, beekeeper documentation, macroscopic characteristics such as sugar crystals or starch residues, and sensorial evaluation, to support authenticity assessment and resolve ambiguous pollen findings.

### 3.6. Critical Synthesis of Conventional Analytical Approaches

The conventional analytical approaches reviewed in this section form the methodological backbone of contemporary honey authentication and remain indispensable for regulatory enforcement, quality control and legal substantiation. Physicochemical testing, spectroscopy, chromatography, stable isotope analysis and melissopalynology each address distinct aspects of honey composition, origin and processing, and together constitute a multi-layered analytical toolbox that has evolved in response to historically dominant fraud practices [12].

Across methods, a consistent pattern emerges: conventional approaches are highly effective when adulteration produces detectable deviations from established reference ranges but increasingly constrained when fraud strategies exploit natural variability, compositional convergence and system-level vulnerabilities. Physicochemical parameters remain essential for compliance assessment but lack resolution for subtle or adaptive manipulation. Spectroscopic techniques offer rapid and scalable screening yet depend critically on calibration design and dataset representativeness. Chromatographic methods provide molecular specificity, but face challenges related to overlap, cost and throughput. Stable isotope analysis delivers robust evidence for classical syrup adulteration, while its interpretative power becomes context-dependent under conditions of baseline variability and blending. Melissopalynology contributes valuable origin-related information but is intrinsically limited by biological variability, processing effects and interpretative subjectivity [5].

Importantly, the limitations observed across these methods are not primarily technical shortcomings, but rather reflect a mismatch between marker-centred analytical paradigms and an adulteration landscape that increasingly operates within the bounds of apparent analytical normality.

This synthesis highlights a central implication for honey authentication: no single conventional method can reliably resolve all relevant fraud scenarios. Analytical robustness increasingly depends on method integration, contextual interpretation and the use of complementary datasets rather than on the refinement of individual techniques alone [5,12]. While multi-method approaches improve resilience, they also increase analytical complexity, cost and interpretative burden, particularly in routine control settings.

Within this context, conventional analytical approaches should be understood as providing a necessary but not sufficient foundation for future authentication strategies. Their strengths lie in quantification, standardisation and legal defensibility; their limitations arise where fraud exploits biological variability, processing-induced decoupling of markers, or system-level features beyond the reach of composition-based analysis. Recognising these boundaries is essential to avoid overinterpretation of analytical results and to maintain scientific and regulatory credibility [29].

These constraints do not diminish the value of conventional methods but rather delineate the space in which advanced and complementary approaches may contribute additional resolution. The following section therefore explores emerging analytical frameworks that extend beyond single-marker paradigms, including omics-based profiling, data integration and system-oriented strategies, with the aim of addressing vulnerabilities that remain unresolved by conventional methodologies alone. As illustrated in Figure 3, the limitations of single-layer analytical approaches underline the need for integrated, multi-layer authentication strategies, which are addressed in detail in the subsequent section on omics-based approaches.

## 4. Omics-Based Analytical Approaches for Honey Authentication

This section synthesises omics-based analytical approaches as advanced, escalation-level tools for honey authentication, with Table 4 summarising their analytical scope, strengths and limitations.

Omics-based analytical approaches comprise high-dimensional molecular profiling techniques that characterise biological and chemical systems at the levels of genes, proteins, metabolites and microbial communities. In honey authentication, these methods enable detection of biological and compositional signatures that are not accessible through conventional physicochemical or spectroscopic analyses, thereby providing system-level information relevant to origin, processing and matrix integrity [4,5,12,56].

Recent reviews identify genomics, proteomics, metabolomics and metagenomic microbiome profiling as the principal omics-based strategies applied to honey authentication. These approaches are primarily implemented via high-throughput sequencing technologies and high-resolution mass spectrometry, complemented by NMR-based metabolite profiling [3,17,23,29,57,58]. Although increasing accessibility has expanded analytical resolution, discriminatory performance varies substantially across matrices and study designs and remains sensitive to biological variability, sampling strategy and the availability of robust reference datasets. High analytical resolution alone therefore does not guarantee unambiguous fraud attribution [6,31,54,58].

From a fraud-control perspective, individual omics layers capture different dimensions of authenticity. Genomic and microbiome-based approaches primarily reflect biological origin and matrix integrity, whereas proteomic and metabolomic methods provide functional and compositional information more directly affected by processing and adulteration [14,23,24,42]. Each approach thus exhibits distinct methodological strengths and interpretative limitations when applied in isolation.

Accordingly, this section synthesises original research published between 2020 and 2025 alongside key review analyses to critically assess the authentication potential, robustness and constraints of omics-based approaches in honey fraud detection. Emphasis is placed on comparative evaluation and methodological complementarity rather than analytical hierarchy, providing the conceptual framework for the detailed discussion of individual omics layers in Section 4.1, Section 4.2, Section 4.3, Section 4.4 and Section 4.5. An overview of the analytical scope, strengths and limitations of the major omics-based approaches relevant to honey adulteration detection is summarised in Table 4.

**Table 4:** Overview of omics-based approaches for honey adulteration detection.

Omics Layer *	Analytical Focus	Strengths	Limitations	Relevance	Evidence Basis
Genomics (DNA barcoding, metabarcoding, metagenomics)	Verification of botanical and geographical origin; plausibility checks for label claims; detection of biological matrix signals (plant- and bee-associated DNA)	High taxonomic resolution; robust to filtration and moderate processing; effective for identifying origin mismatches and biological implausibility	Primer and amplification bias; sequence abundance does not provide a quantitative proxy for nectar contribution; limited ability to detect exogenous sugars or syrup addition.	Plausibility and consistency layer; flags origin inconsistencies but not direct adulteration	[59,60,61,62,63,64,65,66,67]
Proteomics	Integrity assessment via endogenous bee-derived peptides and enzymes; dilution and processing effects (heat, handling, filtration)	Processing-sensitive; supports dilution detection through depletion of native enzymes/proteins; functional insight into honey integrity	Very low protein abundance; protocol-dependent extraction; limited taxonomic scope; no direct identification of adulterant type	Supportive integrity layer for dilution and processing-related fraud	[36,68,69,70]
Metabolomics (LC–MS, GC–MS, NMR, volatilomics)	Detection of compositional deviations caused by syrup addition, dilution and processing; secondary support for origin differentiation	High sensitivity to adulteration; pattern-based fingerprints; broad chemical coverage; applicable to targeted and non-targeted workflows	Confounding effects of botanical origin, storage and processing; feature annotation uncertainty; limited mechanistic interpretability of pattern-based classifications.	Primary analytical layer for honey adulteration detection	[12,29,46,71,72,73,74,75,76,77,78,79,80,81,82,83,84,85,86,87,88]
Metagenomic microbiome profiling	Assessment of biological integrity via microbial community structure; detection of biological dilution and matrix disruption	Sensitive to loss of biological complexity; identifies biologically implausible samples even if chemically compliant	Strongly influenced by geography, handling and storage; limited standardisation; cannot specify adulterant or mechanism	Screening and plausibility tool for engineered or masked fraud	[3,5,6,63,64,66,89]
Multi-omics integration and data fusion	Cross-layer consistency checks; resolution of ambiguous cases; detection of engineered fraud via orthogonal signals	Reduces single-method blind spots; increases evidentiary confidence when layers agree	Data harmonisation challenges; model opacity; reference dataset dependency; high cost and implementation burden	Strategic escalation tool for complex or disputed cases	[5,6,29,66,74,82,86]

* Table 4 provides a representative overview of omics-based approaches applied in honey adulteration detection, highlighting key methodological layers rather than offering an exhaustive survey of all published studies. Omics-based approaches are most effective when applied in combination with complementary analytical techniques, including conventional chemical analyses and multivariate data interpretation, rather than as stand-alone methods.

### 4.1. Genomics-Based Approaches

Genomics-based approaches in honey authentication exploit residual DNA originating from plants, bees and associated microorganisms that becomes incorporated into the honey matrix during nectar foraging, processing and storage. Unlike metabolomic or spectroscopic techniques, which interrogate chemical composition, genomic methods provide direct biological evidence and are therefore primarily suited to questions of botanical and geographical origin rather than compositional adulteration.

#### 4.1.1. DNA Barcoding and Targeted Plant DNA Analysis

Early applications of genomics in honey authentication relied on DNA barcoding to identify plant taxa contributing to nectar sources. Recent studies demonstrate that even low-cost barcoding strategies can yield robust floral origin information when appropriate markers and curated reference databases are employed. Lobo-Torres et al. [59] showed that targeted plant DNA barcoding enables reliable discrimination of botanical origin in complex honeys, including products from biodiverse tropical regions where classical melissopalynology often lacks resolution.

Beyond presence–absence information, quantitative extensions of plant DNA analysis have been proposed to improve discrimination between mono- and multifloral honeys. Dodd et al. [60] demonstrated that relative plant DNA quantification can strengthen origin assignment by comparing proportional DNA signals across samples. However, the authors also emphasised that DNA abundance does not scale linearly with nectar contribution, reflecting marker-specific amplification bias and differential DNA degradation. These constraints limit the interpretability of genomic data when used as proxies for compositional dominance.

#### 4.1.2. DNA Metabarcoding and Methodological Constraints

DNA metabarcoding has substantially expanded the analytical scope of genomics-based honey authentication by enabling simultaneous detection of multiple plant taxa within a single assay. Environmental DNA (eDNA) metabarcoding approaches have been shown to capture complex botanical and geographical signatures, particularly when plant, bee and microbial markers are analysed in parallel. Pathiraja et al. [61] demonstrated that combined analysis of chloroplast plant markers, insect DNA and bacterial profiles can reveal inconsistencies between declared and biologically supported origin in commercial honeys, including mislabelled monofloral products.

Methodological robustness of metabarcoding workflows critically depends on primer selection and bioinformatic processing. Ranieri et al. [62] performed a systematic in silico evaluation of commonly used metabarcoding primers and showed that taxonomic coverage varies markedly between primer sets, resulting in systematic detection biases. From a fraud-control perspective, such biases represent an important limitation, as incomplete taxonomic recovery may obscure relevant botanical signals or generate false confidence in origin assignment.

#### 4.1.3. Bulk DNA Metagenomics and Multi-Taxonomic Signatures

Bulk DNA metagenomic approaches extend beyond targeted barcoding by capturing the full spectrum of DNA present in honey without prior marker selection. Paluoja et al. [63] demonstrated that shotgun metagenomics enables comprehensive reconstruction of honey’s biological composition, including plants, microorganisms and other biological residues, thereby providing a high-dimensional origin-related signature. Earlier work by Wirta et al. [64] similarly showed that DNA traces from plants, bacteria and fungi collectively explain a substantial proportion of variance between honeys from neighbouring regions with similar vegetation.

Importantly, these studies also indicate that DNA-based discrimination remains informative even in filtered or processed honeys. Wirta et al. [64] observed that pollen removal had only a limited effect on taxonomic recovery, suggesting that genomic signals are retained beyond visible pollen content. This characteristic distinguishes genomics from classical melissopalynology and enhances its applicability under real-world processing conditions.

#### 4.1.4. Geographical Inference and Data-Driven Integration

Several studies have explored the use of genomic data for geographical origin assignment. Khansaritoreh et al. [65] demonstrated that DNA metabarcoding can resolve regional differences in honeys by capturing plant assemblages characteristic of specific landscapes. More recent work has combined genomic data with machine-learning approaches to improve classification performance. Liu et al. [66] showed that metagenomic profiles integrated with supervised learning models can discriminate honey origins with high accuracy, while also highlighting the dependence of model robustness on representative training datasets.

From a reviewer perspective, such approaches should be interpreted as probabilistic classification tools rather than definitive origin proof. Their performance is contingent on reference dataset composition, geographic coverage and analytical consistency, limiting transferability across regions with underrepresented botanical diversity.

#### 4.1.5. Strengths, Limitations and Role Within Authentication Frameworks

Across genomic studies, a consistent limitation concerns interpretability beyond origin attribution. While DNA-based approaches provide high taxonomic resolution, they do not directly quantify nectar contribution and cannot detect exogenous sugar addition or enzymatic modification. As demonstrated across multiple studies [59,61,64], sequence abundance is influenced by amplification bias, DNA degradation and marker choice, precluding reliable compositional inference.

Consequently, genomics functions primarily as a consistency and plausibility tool within honey authentication. It is particularly valuable for verifying declared botanical and geographical origin and for identifying mismatches between labelling claims and biological signatures. However, it cannot replace chemical, isotopic or metabolomic analyses in scenarios dominated by syrup adulteration or processing-induced compositional changes.

In summary, genomics-based approaches provide highly specific biological evidence for origin verification and traceability assessment. Their strength lies in detecting origin inconsistencies rather than compositional manipulation, positioning genomics as a supportive but non-standalone component within integrated, multi-layer authentication frameworks.

### 4.2. Proteomics

Proteomic approaches in honey authentication interrogate protein- and peptide-level signatures originating primarily from bee secretions, nectar-associated plant material and, to a lesser extent, hive- and environment-associated microorganisms. In contrast to genomics, which reflects taxonomic presence, proteomics captures functional and processing-sensitive molecular information, thereby providing complementary evidence relevant to processing history, dilution and certain forms of adulteration.

#### 4.2.1. Protein and Peptide Signatures in Honey Matrices

The protein content of honey is intrinsically low, typically below 0.1%, and is dominated by bee-derived enzymes and peptides introduced during nectar processing. As a result, proteomic workflows in honey authentication rely almost exclusively on peptide-centric readouts generated by LC–MS/MS following enzymatic digestion rather than on intact protein profiling. This analytical constraint defines both the strength and the limitation of proteomics in this context.

Takahashi et al. [36] developed and validated a targeted LC–MS/MS method for quantification of α-glucosidase III, a bee-derived enzyme involved in carbohydrate metabolism, and proposed it as a potential authenticity marker. The study demonstrated that endogenous enzyme levels can serve as indicators of honey integrity, particularly in scenarios involving dilution with sugar syrups, where proportional depletion of bee-derived proteins occurs. Importantly, the authors emphasised that such markers reflect biological contribution rather than chemical composition and must therefore be interpreted relative to appropriate reference ranges.

#### 4.2.2. Functional Relevance and Processing Sensitivity

Proteomic signatures are particularly sensitive to processing-related effects, including heat treatment and dilution. Protein denaturation and degradation lead to reproducible alterations in peptide abundance patterns, enabling indirect assessment of thermal history. Moreover, adulteration with sugar syrups reduces the relative abundance of endogenous proteins, producing characteristic quantitative shifts detectable by peptide-based analysis.

Biochemical characterisation of bee-derived products further supports the functional relevance of protein-level markers. Hsu et al. [90] combined metagenomic and biochemical analyses of bee bread and demonstrated that protein-associated functional components reflect both biological origin and processing context. Although the study focused on bee bread rather than honey per se, its findings are directly relevant for understanding the biochemical origin and variability of proteinaceous components transferred into honey.

#### 4.2.3. Bioactivity-Linked Protein Effects and Interpretative Constraints

Beyond authenticity assessment, several studies have investigated protein-linked bioactivities in honey. Li et al. [68] reported antioxidant, antibacterial and anti-hepatocarcinogenic effects associated with specific honey samples, highlighting the functional relevance of protein- and peptide-associated components. From an authentication perspective, such findings are informative but indirect, as bioactivity reflects cumulative molecular effects rather than discrete authenticity markers.

A critical limitation of proteomics in honey authentication lies in analytical variability and database dependency. Protein extraction efficiency is highly protocol-dependent, and peptide identification relies on reference databases that remain incomplete for many plant and microbial taxa associated with honey. Consequently, confident assignment beyond well-characterised bee proteins is often restricted, limiting taxonomic resolution and cross-study comparability.

#### 4.2.4. Methodological Boundaries and Relation to Other Omics Layers

Methodologically adjacent approaches, such as artificial intelligence applied to NMR data, have been explored for honey recognition [69]. While such studies demonstrate the power of data-driven classification, they do not constitute proteomic analysis per se and are therefore best interpreted as complementary chemometric tools rather than protein-focused methodologies.

From a reviewer perspective, proteomics should not be evaluated in isolation or overextended beyond its evidentiary scope. It does not provide direct information on botanical origin and cannot detect exogenous sugars at the molecular level. Its primary contribution lies in detecting processing-related changes and supporting dilution assessment through relative depletion of endogenous proteins.

#### 4.2.5. Role of Proteomics Within Authentication Frameworks

Overall, proteomics represents a technically demanding but informative omics layer for honey authentication. Its strengths lie in sensitivity to processing, dilution and biological integrity, while its limitations include low protein abundance, extraction variability and restricted database coverage. Consequently, proteomics is unlikely to function as a universal standalone authentication tool.

Within integrated authentication frameworks, proteomics provides functional and processing-related evidence that complements genomic origin tracing and metabolomic compositional profiling. When interpreted conservatively and in combination with other omics layers, protein- and peptide-level signatures strengthen mechanistic understanding of authenticity deviations and enhance the robustness of multi-layer honey authentication strategies.

### 4.3. Metabolomics

Metabolomic approaches characterise honey through comprehensive profiling of low-molecular-weight compounds, including sugars, organic acids, amino acids, phenolic compounds and volatiles. Because these metabolites directly reflect compositional integrity, metabolomics is particularly well suited for detecting adulteration and processing-related manipulation, while also contributing to origin differentiation.

#### 4.3.1. Mass-Spectrometry-Based Metabolomics for Adulteration Detection

Untargeted LC-MS-based metabolomics has become a central tool for identifying compositional deviations caused by syrup addition or excessive processing. High-resolution LC-MS/MS profiling of phenolic compounds and related secondary metabolites reveals reproducible shifts in metabolite patterns when authentic honey matrices are diluted or altered. Mostoles et al. [71] and Sarmento et al. [72] demonstrated that polyphenolic fingerprints discriminate authentic honeys from altered products, while also reflecting botanical background.

Rather than relying on individual markers, several studies highlight the superiority of pattern-based interpretation. Silva et al. [73] showed that multi-biomarker HRMS strategies outperform single-compound approaches for identifying deviations in honeydew honeys. Similarly, non-targeted LC-MS workflows enable detection of subtle adulteration effects by capturing thousands of features simultaneously [74]. However, these approaches require rigorous statistical validation to avoid overfitting and false positives.

Elemental metabolomics provides an additional compositional layer. Drivelos et al. [75] demonstrated that elemental fingerprints contribute to discrimination between authentic and non-authentic honeys, although environmental variability limits their standalone interpretability.

#### 4.3.2. Volatilomics as a Sensitive but Context-Dependent Indicator

GC-MS-based volatilomics captures aroma-active compounds that are highly sensitive to compositional changes. Studies consistently show that volatile profiles are altered by dilution and thermal treatment. Sonia et al. [76] and Zhang et al. [77] demonstrated that volatile fingerprints differentiate honeys of different origins, while Abouelenein et al. [78] showed that combined volatile and phenolic profiling enhances discrimination power.

From an adulteration perspective, volatilomics is valuable but context-dependent, as volatile composition is also influenced by storage and processing conditions. Reviews emphasise that volatile markers must therefore be interpreted relative to controlled reference datasets [29].

#### 4.3.3. NMR-Based Metabolomics and Compositional Integrity

NMR spectroscopy provides highly reproducible metabolomic fingerprints and is particularly effective for detecting syrup adulteration and abnormal sugar profiles. Ji et al. [79] demonstrated that ^1^H-NMR combined with machine learning reliably identifies adulteration in wolfberry honey. Additional studies confirm that NMR-based metabolomics detects deviations in sugar ratios, organic acids and minor metabolites across diverse honey types [80,81,82,83].

Compared with LC-MS, NMR offers lower sensitivity for trace-level compounds but superior quantitative robustness, making it well suited for routine adulteration screening and interlaboratory comparability [84].

#### 4.3.4. Interpretative Limits and Role in Fraud Control

Despite its high discriminatory power, metabolomics does not directly identify the adulterant or the mechanism of fraud. Metabolomic deviations may reflect multiple overlapping factors, including botanical variability, processing and storage. Amino acid profiling [85] and combined MS- and spectroscopic approaches [12] illustrate that strong classification performance must be interpreted cautiously and within a well-defined reference framework.

Overall, metabolomics represents the most compositionally explicit omics layer for honey adulteration detection. Its primary strength lies in sensitivity to syrup addition, dilution and processing effects, while its limitations relate to interpretability and standardisation. Consequently, metabolomics provides robust evidence for compositional inconsistency, but achieves maximum forensic value when integrated with genomic and proteomic data that contextualise biological origin and processing history.

### 4.4. Metagenomic Microbiome Profiling

Metagenomic microbiome profiling examines microbial DNA signatures embedded in the honey matrix and provides a system-level perspective on biological integrity. While microbial communities are often discussed in ecological or origin-related contexts, their primary relevance for honey adulteration lies in detecting biological dilution and matrix disruption that accompany syrup addition, excessive processing or replacement of authentic honey components.

#### 4.4.1. Microbial Baselines and Biological Dilution Effects

Authentic honey contains characteristic microbial DNA originating from nectar, pollen, bees and the hive environment. Shotgun metagenomic analyses demonstrate that these microbial assemblages form reproducible background signatures. Paluoja et al. [63] showed that multi-kingdom DNA profiles, including bacteria and fungi, persist across processing steps and therefore constitute a stable biological baseline. Wirta et al. [64] similarly demonstrated that microbial DNA contributes substantially to sample differentiation, even when botanical signals overlap.

From an adulteration perspective, these findings are critical because the addition of exogenous sugar syrups reduces the relative abundance and complexity of honey-specific microbial DNA. Such shifts cannot be explained by botanical variability alone and therefore serve as indicators of biological dilution.

#### 4.4.2. Processing- and Adulteration-Related Perturbations

Microbiome profiling is particularly sensitive to interventions that alter the biological matrix without producing immediately diagnostic chemical signals. Hsu et al. [90] demonstrated that combined metagenomic and biochemical analyses of bee-derived matrices reveal functional and microbial changes associated with processing history. Although focused on bee bread, the study illustrates a transferable principle: biological processing leaves detectable microbial traces that are attenuated or distorted by dilution and manipulation.

Data-driven approaches further support this interpretation. Liu et al. [66] showed that metagenomic profiles integrated into machine-learning models can discriminate authentic honeys from altered products. Importantly, this discrimination reflects system-level biological disruption rather than identification of specific adulterants, highlighting microbiome profiling as a deviation-detection tool.

#### 4.4.3. Interpretative Constraints and Methodological Limits

Despite their sensitivity, microbiome-based approaches face significant interpretative constraints. Microbial communities are influenced by geography, climate, storage and handling conditions, complicating the establishment of universal reference baselines. Reviews and methodological analyses emphasise that microbiome-derived discrimination must therefore be interpreted within tightly defined reference frameworks [3,5].

Walker et al. [6] cautioned that high-dimensional biological datasets may yield strong classification performance even when causal interpretation is weak. Consequently, microbiome profiling does not provide direct evidence of the type or mechanism of adulteration, but rather signals loss of biological authenticity.

Contextual datasets combining microbiological and chemical information further support cautious interpretation. Huyop et al. [89] illustrated that microbial and molecular datasets can complement compositional analyses, but do not substitute for targeted adulterant detection.

#### 4.4.4. Role Within Adulteration-Focused Authentication Frameworks

Within honey adulteration control, metagenomic microbiome profiling functions as a biological plausibility and screening tool. Its primary value lies in identifying samples where biological complexity is inconsistent with genuine honey, even when chemical parameters appear compliant.

Accordingly, microbiome profiling strengthens adulteration detection when combined with metabolomic and proteomic analyses that identify compositional and functional changes. Used conservatively and in a multi-layer framework, microbiome-derived signals help prioritise samples for further investigation rather than serving as standalone proof of fraud.

### 4.5. Multi-Omics Integration and Data Fusion

Multi-omics integration seeks to combine complementary analytical layers—genomic, proteomic, metabolomic and microbiome-derived data—to strengthen honey authentication and adulteration detection. Rather than increasing analytical complexity for its own sake, the rationale for data fusion lies in addressing the intrinsic limitations of individual omics approaches, particularly in fraud scenarios designed to evade single-method detection.

#### 4.5.1. Rationale for Multi-Omics Approaches in Adulteration Detection

Single-omics methods capture distinct but partial aspects of honey authenticity. Genomics and microbiome profiling primarily reflect biological origin and matrix integrity, while metabolomics and proteomics provide chemically and functionally explicit information on composition and processing. In isolation, each layer remains vulnerable to ambiguity: biological signals may persist after dilution, and chemical fingerprints may be engineered to mimic authenticity thresholds.

Multi-omics strategies aim to resolve such ambiguities by integrating orthogonal information streams. Navarro-Herrera et al. [86] demonstrated that explicit data fusion of GC-Orbitrap-HRMS metabolomics with complementary analytical techniques enhances discrimination between monofloral, multifloral and compositionally altered honeys. Importantly, the added value did not arise from increased marker density alone, but from the consistency of signals across independent analytical domains.

#### 4.5.2. Data-Driven Integration and Machine Learning

Several studies have explored the use of machine learning to integrate high-dimensional omics datasets for honey authentication. Liu et al. [66] combined metagenomic features with supervised learning models to classify honey origin, illustrating how biological and statistical integration can outperform single-layer analyses. Similarly, Chahal et al. [74] demonstrated that robust feature selection from non-targeted LC-MS data improves classification stability, a principle directly transferable to multi-omics contexts.

Quantitative NMR studies further support integrative strategies. Burton et al. [82] showed that multivariate statistical modelling of metabolomic fingerprints yields reproducible discrimination across large sample sets, providing a stable backbone for integration with other omics layers.

From an adulteration perspective, such data-driven integration is particularly valuable for detecting engineered fraud, where compositional manipulation is calibrated to pass individual analytical tests but fails to produce coherent multi-layer consistency.

#### 4.5.3. Reviews and Conceptual Frameworks

Recent reviews emphasise that multi-omics integration represents an emerging but still heterogeneous field. Zhang et al. [5] highlighted that while multi-omics approaches improve classification performance, their implementation is limited by data harmonisation, reference availability and interpretative transparency. Similarly, Kaldeli et al. [29] discussed volatilomics within broader food authentication frameworks and stressed that integration should be guided by fraud hypotheses rather than by analytical completeness.

These reviews converge on a key principle: integration without conceptual clarity risks generating highly discriminative but poorly interpretable models, a concern that is particularly relevant in regulatory and forensic contexts.

Despite the growing interest in multi-omics integration for food authentication, it is essential to critically reflect on the current methodological and practical limitations of such approaches. Challenges related to data harmonisation, cross-platform integration, model interpretability, and regulatory applicability remain substantial and are increasingly discussed in the broader multi-omics literature [5,58].

From a regulatory perspective, these constraints underscore that omics-based methods should not be interpreted as standalone proof of adulteration, but rather as complementary evidence within integrated authentication frameworks. Their strength lies in identifying inconsistencies and plausibility gaps, while confirmatory interpretation requires contextualisation within established analytical and regulatory criteria.

Accordingly, the added analytical value of multi-omics integration must be balanced against increased complexity, cost and standardisation requirements, particularly in routine control settings.

#### 4.5.4. Methodological and Interpretative Limitations

Despite their promise, multi-omics approaches face substantial challenges. Differences in data structure, scale and noise between omics layers complicate integration, while machine-learning models may amplify artefacts if training datasets are unbalanced or insufficiently representative. Walker et al. [6] explicitly warned that increasingly complex analytical outputs may obscure rather than clarify authenticity decisions if transparency and causality are not preserved.

From a fraud-control perspective, the central risk of multi-omics integration lies not in analytical failure, but in overinterpretation. High classification accuracy does not necessarily equate to mechanistic understanding or legal defensibility, particularly when decision boundaries are driven by opaque model architectures.

#### 4.5.5. Role Within Adulteration-Focused Authentication Strategies

Within honey adulteration control, multi-omics integration should therefore be viewed as a strategic escalation tool, applied selectively when single-omics analyses yield ambiguous or conflicting results. Its primary strength lies in detecting cross-layer inconsistencies, such as chemically compliant samples that lack biological complexity, or biologically plausible samples with anomalous compositional fingerprints.

Used conservatively, multi-omics data fusion enhances confidence in adulteration assessment by reinforcing conclusions across independent analytical dimensions. However, it does not replace well-validated single-method assays and should be embedded within transparent, hypothesis-driven authentication frameworks [5].

Taken together, the omics-based approaches discussed in this section represent advanced, escalation-level tools within a broader, tiered framework for honey fraud authentication. Their analytical value lies not in routine application, but in their strategic integration with conventional and advanced methods when compositional ambiguity, adaptive fraud strategies or enforcement-relevant questions cannot be resolved at earlier analytical levels (Figure 3) [58].

Operationalising integrated governance requires more than analytical harmonisation alone. Practical mechanisms include standardised reporting formats for non-compliant findings, shared reference databases for compositional and biological authenticity profiles, and clearly defined protocols for data exchange between competent authorities. Such structures support cross-regional comparability while reducing duplication of effort and analytical fragmentation [6,38].

Importantly, governance frameworks must also address forms of honey fraud that remain largely inaccessible to laboratory testing, including certification misuse, documentation fraud and manipulation of digital traceability systems. In these cases, analytical results need to be complemented by audit-based controls, supply chain verification and coordinated policy instruments that link laboratory evidence with administrative and enforcement actions [10].

From a practical perspective, multi-omics integration is most effective when applied to clearly defined fraud questions rather than as a universal analytical objective. For example, suspected botanical or geographical origin misrepresentation can be addressed by combining genomic plausibility checks with metabolomic profiling, allowing inconsistencies between declared origin and compositional signatures to be identified. In contrast, suspected syrup adulteration primarily benefits from metabolomics-based pattern analysis, with proteomic or microbiome-derived signals providing supportive evidence of dilution or matrix disruption, consistent with the decision-oriented framework illustrated in Figure 2.

For regulatory applicability, data harmonisation and model interpretability are critical constraints. Multi-omics workflows require standardised preprocessing, reference datasets and transparent decision rules to ensure reproducibility across laboratories. While complex machine learning models may enhance classification performance, their regulatory acceptance depends on traceable feature relevance and explainable outputs that can be linked to defined authenticity criteria [45,58].

## 5. Conclusions and Perspectives

Honey authentication remains a complex and evolving challenge that cannot be resolved through single analytical techniques or isolated methodological advances. As synthesised across Section 2, Section 3 and Section 4, both conventional physicochemical methods and advanced omics-based approaches provide complementary, rather than hierarchical, contributions to authenticity assessment. Their combined use is essential to address the increasing sophistication of adulteration strategies and the growing convergence between authentic and manipulated honey profiles [5,19,35].

Conventional analytical techniques continue to represent indispensable pillars of routine honey control due to their scalability, cost-effectiveness and regulatory familiarity [51,55]. At the same time, omics-based methods offer enhanced resolution in cases where compositional manipulation, signature convergence or system-aware adulteration strategies cannot be reliably resolved at earlier analytical levels [5,6]. Importantly, these advanced approaches extend, rather than replace, established methods and must be interpreted within clearly defined evidentiary boundaries [3,12,58].

Different omics domains contribute distinct and complementary insights into honey authenticity. Genomics and transcriptomics primarily support botanical origin verification and detection of plant-derived adulterants [3,61], whereas metabolomics and proteomics provide broader compositional and process-related information [17,29]. No single omics layer provides comprehensive forensic certainty, and analytical strength emerges primarily from the contextual integration of multiple data streams rather than from hierarchical method escalation.

Taken together, the omics-based approaches discussed in this section demonstrate their greatest value when applied as targeted escalation tools for complex or ambiguous cases that cannot be resolved using conventional analytical workflows alone (Figure 3) [5,19]. Their application therefore requires careful alignment with defined decision thresholds, transparent interpretation frameworks and regulatory relevance [58].

Operationalising integrated honey authentication frameworks requires more than analytical harmonisation alone. Effective implementation depends on coordinated governance structures, harmonised reference datasets, transparent decision criteria and clear attribution of responsibilities across laboratories, competent authorities and enforcement bodies [6,38]. Without such systemic alignment, even technically advanced analytical outputs risk limited legal defensibility and reduced impact in enforcement actions [58].

The added analytical value of multi-layer and multi-omics integration must therefore be balanced against practical constraints, including infrastructure requirements, analytical costs, data interpretation complexity and long-term sustainability [19,20]. Advanced analytical strategies should be deployed proportionately, in line with clearly defined risk profiles and regulatory objectives, rather than as universal screening tools [5].

Significant global disparities in analytical capacity further complicate the implementation of high-resolution authentication strategies. Many regions with substantial honey production or export activity lack access to advanced analytical infrastructure, reference databases or specialised expertise [19,20,21]. These structural inequalities underscore the need for scalable and inclusive authentication models that avoid reinforcing existing asymmetries in regulatory oversight.

Tiered and hub-based authentication frameworks offer a pragmatic response to these challenges [20,21]. Such models combine broad, low-cost screening at primary control levels with targeted escalation to specialised reference laboratories equipped for advanced analytical investigation [19,58]. By preserving proportionality and resource efficiency, these approaches enable meaningful fraud detection while maintaining legal robustness and international comparability [20,58].

Ultimately, resilient honey authenticity assurance depends on the integration of analytical innovation with governance, transparency and international cooperation [6,10]. By embedding advanced analytical tools within structured, interpretable and scalable decision frameworks, honey authentication can move beyond isolated method performance towards system-level robustness [5,58]. This integrated perspective is essential to support credible enforcement, consumer trust and fair trade in increasingly complex global honey supply chains [9].

## Data Availability

No new data were created or analyzed in this study. Data sharing is not applicable to this article.

## Abbreviations

The following abbreviations are used in this manuscript:

AI	Artificial Intelligence
C3	C3 photosynthetic pathway (Calvin cycle plants)
C4	C4 photosynthetic pathway (Hatch–Slack pathway plants)
CSIA	Compound-Specific Isotope Analysis
DNA	Deoxyribonucleic Acid
δ^13^C	Delta carbon-13 isotope ratio
EC	Electrical Conductivity
eDNA	Environmental DNA
FTIR	Fourier Transform Infrared Spectroscopy
GC–MS	Gas Chromatography–Mass Spectrometry
GC-Orbitrap-HRMS	Gas Chromatography coupled to Orbitrap High-Resolution Mass Spectrometry
HMF	Hydroxymethylfurfural
HPLC	High-Performance Liquid Chromatography
HRMS	High-Resolution Mass Spectrometry
IRMS	Isotope Ratio Mass Spectrometry
LC–MS	Liquid Chromatography–Mass Spectrometry
LC–MS/MS	Liquid Chromatography–Tandem Mass Spectrometry
MIR	Mid-Infrared
MS	Mass Spectrometry
NIR	Near-Infrared
NMR	Nuclear Magnetic Resonance
^1^H-NMR	Proton Nuclear Magnetic Resonance
pH	Potential of Hydrogen
UV–Vis	Ultraviolet–Visible Spectroscopy
